# God and the Girl

**DOI:** 10.1007/s11406-020-00295-2

**Published:** 2020-11-23

**Authors:** Benoit Gaultier

**Affiliations:** Philosophisches Seminar, University of Zurich, Zürichbergstrasse 43, 8044 Zürich, Switzerland

**Keywords:** Agnosticism, Atheism, Theism, Wishful thinking

## Abstract

**Abstract**

Imagine you are an agnostic who wants to maximise your chances of getting the right answer to the question whether God exists. I show that theism and atheism are not on an epistemic par with one another because, under certain possible epistemically neutral conditions, the rational thing for you to do from a purely epistemic point of view would be to bet on the atheist’s judgement that God doesn’t exist rather than on the theist’s judgement that God does exist.

## The Girl

Let’s imagine three students, Mary, Tom and Peter. Tom and Peter are inseparable friends who both know Mary but never spent any one-on-one time with her, and who both know exactly the same facts about her. (We can suppose that when one of them learns a new fact about Mary, he quickly communicates it to the other.) Tom believes that Mary is in love with him while Peter believes that this is not the case.^1^ (This often leads them to have exchanges of this type: ‘Peter, don’t you see what happens when Mary and I meet? – Well, what I see is that nothing’s happening on her side.’)

Let’s now introduce a fourth individual, Diane, who learns all these facts about Mary, Tom and Peter. Before learning these facts, Diane neither believed that Mary is in love with Tom, nor that she is not in love with him, nor that one of these two options is more likely than the other. Let’s imagine that there is another fact that she learns: Tom desires that Mary is in love with him and Peter also desires that she is in love with Tom, or at least does not desire that this is not the case.

Suppose now that Diane has to bet on whether Mary is in love with Tom. If her goal is to bet on the right answer, we clearly have the intuition that it would be rational for her to rely on Peter’s judgement rather than on Tom’s on the grounds that the latter is more likely to be biased than the former. More precisely, if Diane knows the human tendency to engage in wishful thinking (in particular, to engage in it in cases where the evidence does not obviously indicate that what is desired is not the case) and learns that Tom desires that Mary is in love with him and that Peter does not desire the opposite, then Diane’s belief that Peter’s judgement on the issue in question is less likely to be biased than Tom’s clearly is correct if based on the following reasoning: when, on a given matter, what a subject believes to obtain is also what she desires to obtain, while, on that matter, this is not the case for another subject in the same evidential situation, there is, on the basis of these facts we know about them, a chance that the human tendency to engage in wishful thinking explains the former subject’s belief while there is no chance that this tendency explains the latter subject’s belief (since what she believes to obtain is not something she desires to obtain). And this means that, on the basis of the facts we know about these two subjects, there is a chance that the former’s judgement is biased while there is no chance that the latter’s judgement is so. Now, the fact that Peter’s judgement is less likely to be biased—i.e. determined by truth-irrelevant factors—on this issue than Tom’s means that the latter is less likely to be reliable or truth-conducive on this issue than the former. So, if Diane wants to maximise her chances of betting on the right answer as to whether Mary is in love with Tom, it seems that the rational option, given the facts that she knows, is to rely on Peter’s judgement rather than on Tom’s, and so to bet on ‘Mary is not in love with Tom’.

Maybe one could object that the feeling that Tom has, when they meet Mary, that she is in love with him makes his evidential situation different from Peter’s who, on his side, does not have this feeling. As the consequence, the conclusion that Diane should bet on Peter’s judgement is problematic since the reasoning from which it follows is based on the idea that the two friends are in the same evidential situation. Whether this objection can be resisted or not, let’s consider a variant of our case that is not subject to this objection because this variant is neutral on whether Tom’s distinctive feeling makes his evidential situation different from Peter’s. Suppose that Diane knows all the facts that she knows in our original case, except for the fact that Tom and Peter are, on the issue in question, in a similar evidential situation. Not knowing anything of their respective evidential situations but still knowing that Tom desires and believes that Mary is in love with him while Peter believes but does not desire the opposite, the rational option for Diane, given what she knows, is once again to bet on Peter’s judgement rather than on Tom’s because the latter is more likely to be biased, and so less likely to be reliable, than the former. Indeed, the respective evidential situations of the subjects involved does not need to intervene for the reasoning mentioned above to be correct: when, on a given matter, what a subject S1 believes to obtain is also what she desires to obtain, while, on that matter, this is not the case for another subject S2, there is, on the basis of these facts, a chance that the human tendency to wishful thinking explains S1’s belief while there is no chance that this tendency explains S2’s belief (since what she believes to obtain is not something she desires to obtain)—which means that there is a chance that S1’s judgement is biased while there is no chance that S2’s is so.^2^

Things differ however when it comes to a second variant of our case, in which Diane knows Tom’s and Peter’s opposing beliefs on whether Mary is in love with Tom (and either knows that Tom and Peter are in a similar evidential situation or is completely ignorant of their respective evidential situations) but is not informed of Tom’s and Peter’s respective desires. Indeed, in this case, there is no reason to think that it is more likely that Tom’s judgement is biased by a desire that Mary is in love with him than Peter’s judgement is biased by a desire that she is not. So it would not be rational for Diane to rely on Peter’s judgement rather than on Tom’s in this case in which she does not know their respective desires.

Finally, let’s imagine a third variant of our case, in which Diane not only knows Tom’s and Peter’s opposing beliefs and is completely ignorant of their respective evidential situations, but also receives this information about their respective conative attitudes: the probability that Tom desires that Mary is in love with him is higher than the probability that Peter desires that she is not. Once again, the rational option for Diane would be to bet on Peter’s judgement rather than on Tom’s because, given the fact that the probability that Tom desires that Mary is in love with him is higher than the probability that Peter desires the opposite, Peter’s judgement is less likely to be biased, and so more likely to be reliable, than Tom’s.

The whole of the foregoing can be summarised as follows:

When one wants to maximise one’s chances of choosing the right answer to the question whether p and one’s only evidence on this issue is that a subject S1 desires and believes that p and another subject S2 believes that ~p but does not desire that ~p, the rational option clearly is to bet on S2’s judgement rather than on S1’s because the former is less likely to be biased, and so more likely to be reliable, than the latter. The same goes if one’s evidence is that S1 believes that p, S2 believes that ~p, and the probability that S1 desires that p is higher than the probability that S2 desires that ~p. But when one knows S1’s and S2’s respective beliefs as to whether p but has no idea at all of their conative attitudes, it would not be rational for one, in this evidential situation, to rely on S2’s judgement rather than on S1’s because in this case there is no reason to think that the latter is more likely to be biased, and so less likely to be reliable, than the former.^3^

## God

Let’s now turn to a case that is structurally similar to our original case, but no longer about Mary and whether she is in love with Tom, but about God and whether he exists. Let’s imagine that two friends, Tom and Peter, have discussed whether God exists at length over many years. In their discussions, they cover all the arguments, facts and experiences they take to be relevant to the question of whether God exists. However, Tom and Peter disagree about whether God exists, and their disagreement is not purely verbal: by ‘God’ they both mean a perfectly good and omniscient being who created the Universe, due to whom this truly is the best of all possible worlds, who has infinite love for each of us, and whose existence implies that our biological death does not mean the end of our existence but may be followed by our everlasting happiness. While Tom believes that God exists, Peter believes that this is not the case. (This often leads them to have exchanges of this type: ‘Peter, don’t you feel the presence and love of God when you look at the way things are in the Universe? – Well, no, I don’t feel anything like that, quite the contrary in fact.’)

Let’s once again introduce a fourth character, Diane, who learns these different facts about Tom and Peter, neither of whom she knew anything about before. Before she learned these facts, Diane was perfectly agnostic concerning the existence of God as both Tom and Peter conceive him: before learning these facts, she neither believed that he exists, nor that he does not, nor that one of these two alternatives is more likely than the other. Let’s imagine that, as in our original case, there is another fact that she learns: Tom desires that God exists and Peter also has this desire, or at least does not desire the opposite.

Suppose now that Diane has to bet on whether such a God exists and wants to maximise her chances of betting on the right answer (setting aside any Pascalian pragmatic considerations). Because there is no relevant difference between Diane’s evidential situation in this case and her evidential situation in our original case in which it clearly seems that the rational option is to bet on Peter’s judgement, the rational option here also is to bet on Peter’s judgement on the grounds that it is less likely to be biased than Tom’s.

Just as with our original case about Mary, maybe one could object that Tom’s feeling of the presence and love of God makes his evidential situation different from Peter’s who, on his side, does not have this feeling (and even has an opposing feeling). As a consequence, the objection once again goes, the conclusion that Diane should bet on Peter’s judgement is problematic since the reasoning from which it follows is based on the idea that the two friends are in the same evidential situation. But, just as in the previous section, a variant in which Diane does not know Tom or Peter’s respective evidential situations on the matter of God’s existence is not subject to this objection—so that in this variant, which is structurally similar to our second case about Mary, Diane should bet on Peter’s judgement just as she should do in the second case.

The same goes for a case, structurally similar to our fourth case, in which Diane is informed that the probability that Tom desires that God exists is higher than the probability that Peter desires the opposite. Because there is no relevant difference between Diane’s evidential situation in these cases about God’s existence and her evidential situation in the corresponding cases about Mary’s feelings for Tom, the rational option in these new cases would also be to bet on Peter’s judgement on the grounds that it is less likely to be biased, and so more likely to be reliable, than Tom’s.

What now about a case in which Diane knows that Tom believes that God exists and that Peter believes the opposite and is completely ignorant of their respective evidential situations, but is not informed of Tom’s and Peter’s respective conative attitudes, nor receives the information that the probability that Tom desires that God exists is higher than the probability that Peter desires the opposite?

An important difference appears between this case and our third case. Let’s suppose that Diane has noted the empirical fact that the percentage of believers who would prefer that God, as both Tom and Peter conceive him, does not exist is much lower than the percentage of atheists who would prefer that he does exist (which makes it much more frequent to hear atheists lamenting his non-existence than believers lamenting his existence). It would be intuitively correct for Diane, if she has noted this empirical fact and also knows the properties that Tom and Peter both attribute to God and so what they both take to follow from his existence, to judge that the probability that Tom desires that God exists is higher than the probability that Peter desires that he does not. Diane is therefore in an evidential situation similar to that of our fourth case, in which her evidence was that (a) the probability that Tom desires that Mary is in love with him is higher than the probability that Peter desires the opposite, and that (b) Tom believes that Mary is in love with him while Peter believes the opposite. So if Diane wants to maximise her chances of betting on the right answer to the question whether such a God exists, the rational option, just as in this fourth case, is to bet on Peter’s judgement rather than on Tom’s, because Tom is more likely to be biased than Peter.

This point can be generalised in the following way:

- If one is perfectly agnostic concerning the existence of God as both Tom and Peter conceive him; and
- if one then learns that there are as many atheists as believers concerning the existence of such a God; and
- if one does not know anything about these atheists and believers, other than that humans tend to engage in wishful thinking, and that the percentage of believers who would prefer that such a God does not exist is much lower than the percentage of atheists who would prefer that he exists;
- then the rational option if one wants to maximise one’s chances of choosing the right answer to the question whether such a God exists is to bet on the atheists’ judgement because it is less likely to be biased, and so more likely to be reliable, than the believers’ judgement.

(Let’s note that this argument does not just apply to the question of whether God exists. It can apply to any proposition p about which one is perfectly agnostic and about which one comes to know a) that there are as many people who believe that p as people who believe that ~p, and b) that the percentage of the former who would prefer that ~p is lower than the percentage of the latter who would prefer that p (while not knowing anything else about these people). Let’s take for example the proposition that hydroxychloroquine works against SARS-CoV-2, and let’s suppose that Diane, who is perfectly agnostic concerning this question, learns a) that those who believe that it works are as numerous as those who believe the contrary, and that b) the percentage of the former who would prefer that hydroxychloroquine does not work is lower than the percentage of the latter who would prefer that this cheap drug works (while not knowing anything else about those people). In this evidential situation, the rational option for Diane, if she wants to maximise her chances of choosing the right answer to this question, is to bet on the judgement of those who believe that hydroxychloroquine does not work because their judgement is less likely to be biased, and so more likely to be reliable, than the judgement of those who believe the contrary.)

It might be claimed that while this argument is sufficient to show that theism and atheism are not on an epistemic par, it does not show that betting on the atheist’s belief would be more rational because, as a matter of fact, there are not as many atheists as believers. This is indisputably true, but maybe the scope of this argument can be made wider. Suppose that we modify our initial scenario in the following way: Peter still believes, but does not desire, that Mary is not in love with Tom, but in addition to Tom, another student, Roger, desires and believes that Mary is in love with Tom; and just as with Tom and Peter, this is the only thing that Diane knows about Roger. In this situation, Diane’s only evidence regarding whether Mary is in love with Tom is that two subjects S1 and S2 desire and believe that this is the case, and another subject S3 believes, but does not desire, that the contrary is true. It still seems that it would be rational for Diane, if she wants to maximise her chances of choosing the right answer to the question at issue, to rely on Peter’s judgement rather than on Tom’s and Roger’s on the grounds that the latter are more likely to be biased, and so less likely to be reliable, than the former. If this is correct, then in virtue of the structural similarity we have highlighted between the case about Mary and whether she is in love with Tom, and the case about God and whether he exists, it directly follows that the conclusion (d) still holds when what one comes to know that there is at least one atheist for every two believers—which is closer to the present distribution of atheists and believers.^4^

## Plantinga

Even though I have not claimed, in a Freudian fashion, that belief in God is probably false because it probably results from wishful thinking, can Alvin Plantinga’s way of arguing against this Freudian claim refute our point that in the evidential situation in which one finds oneself when conditions (a), (b), and (c) are satisfied, it is more likely that belief in God is false? Here is his reasoning:

Even if it were established that wish-fulfillment is the source of theistic belief, however, that wouldn’t be enough to establish that the latter has no warrant. It must also be established that wish-fulfillment in this particular manifestation is not aimed at true belief. The cognitive design plan of human beings is subtle and complicated; a source of belief might be such that in general it isn’t aimed at the formation of true belief, but in some special cases it is. So perhaps this is true of wish-fulfillment; in general, its purpose is not that of producing true belief, but in this special case precisely that is its purpose. Perhaps human beings have been created by God with a deep need to believe in his presence and goodness and love. Perhaps God designed us that way in order that we come to believe in him and be aware of his presence. Perhaps this is how God has arranged for us to come to know him. If so, then the particular bit of the cognitive design plan governing the formation of theistic belief is indeed aimed at true belief, even if the belief in question arises from wish-fulfillment. Perhaps God has designed us to know that he is present and loves us by way of creating us with a strong desire for him, a desire that leads to the belief that in fact he is there. (Plantinga 2000: 197)

Let’s admit, with Plantinga, that if God existed, then the belief that he exists would not only be true but produced in a truth-conducive way by truth-conducive wishful thinking. The truth of this conditional does not raise the probability that God exists, and so does not raise the probability that Tom’s belief has been produced in a truth-conducive way. Therefore, to admit that if God existed the belief that he exists would be produced in a truth-conducive way does not change the fact that it is rational, in the kind of evidential situation in which Diane is in our last case, to bet on the atheists’ judgement rather than on the believers’ if one wants to maximise one’s chances of choosing the right answer to the question whether God exists. This means that theism and atheism are not on an epistemic par with one another. This is a conclusion we can reach without, contrary to Freudians, presupposing in any way the truth of atheism nor endorsing any view of what are, in fact, the origins of religious belief.

## Footnotes

1 Since it has been stipulated that they both know the same facts about Mary, it follows that neither Tom’s nor Peter’s opposing beliefs on whether Mary loves Tom amounts to knowledge—i.e. that neither of them knows the truth regarding this issue.

2 Thanks to an anonymous referee for *Philosophia* for having raised the objection addressed in this paragraph, and for suggesting I make it more explicit than in a previous version of this section why Diane would be correct to bet on Peter’s judgement. More generally, due to this referee’s remarks, the present section importantly improved.

3 It is interesting to note that, contrary to what it may seem, these conclusions are irrelevant to the epistemological debate concerning the epistemic consequences of a disagreement between epistemic peers. Indeed, having set aside the original version of our case in which the subjects involved were supposed to be in a similar evidential situation, these conclusions just apply to cases in which one is ignorant of the respective reliability and evidential situation of the subjects involved. Nevertheless, something can be said about the relation between cases of disagreement between epistemic peers and our original case: this case was supposed to be a case of disagreement between *evidential* peers whose respective reliability when it comes to processing their identical evidence on the matter at issue is initially unknown to us. The conclusion to be drawn from such a case is the following: if the subjects truly are *evidential* peers on the matter at issue, and the only other thing we know about them is that what one of them believes to obtain is also what she desires to obtain while this is not the case for the other, this (non*-*conclusively) speaks in favour of their not being *epistemic* peers, because it means that one of them is more likely to be biased, and so less likely to be *reliable*, than the other.

4 Thanks to the same anonymous referee for *Philosophia* whose incisive remarks led me to clarify, in the last two paragraphs of this section, the scope of the argument developed above.
